# Constructing phylogenetic trees for microbiome data analysis: A mini-review

**DOI:** 10.1016/j.csbj.2024.10.032

**Published:** 2024-10-24

**Authors:** Ruitao Liu, Xi Qiao, Yushu Shi, Christine B. Peterson, William S. Bush, Fabio Cominelli, Ming Wang, Liangliang Zhang

**Affiliations:** a Department of Population and Quantitative Health Sciences, School of Medicine, Case Western Reserve University, 10900 Euclid Avenue, Cleveland, 44106, OH, United States; b Weill Cornell Medicine, Cornell University, 1300 York Ave, New York, 10065, NY, United States; c The University of Texas MD Anderson Cancer Center, 1515 Holcombe Blvd, Houston, 77030, TX, United States; d Department of Pathology, School of Medicine, Case Western Reserve University, 10900 Euclid Avenue, Cleveland, 44106, OH, United States; e Case Digestive Health Research Institute, Case Western Reserve University, 10900 Euclid Avenue, Cleveland, 44106, OH, United States; f Case Comprehensive Cancer Center, 10900 Euclid Avenue, Cleveland, 44106, OH, United States

**Keywords:** Phylogenetic trees, Alignment, Microbiome, Shotgun sequencing, 16S sequencing

## Abstract

As next-generation sequencing technologies advance rapidly and the cost of metagenomic sequencing continues to decrease, researchers now face an unprecedented volume of microbiome data. This surge has stimulated the development of scalable microbiome data analysis methods and necessitated the incorporation of phylogenetic information into microbiome analysis for improved accuracy. Tools for constructing phylogenetic trees from 16S rRNA sequencing data are well-established, as the highly conserved regions of the 16S gene are limited, simplifying the identification of marker genes. In contrast, metagenomic and whole genome shotgun (WGS) sequencing involve sequencing from random fragments of the entire gene, making identification of consistent marker genes challenging owing to the vast diversity of genomic regions, resulting in a scarcity of robust tools for constructing phylogenetic trees. Although bacterial sequence tree construction tools exist for upstream bioinformatics, many downstream researchers—those integrating these trees into statistical models or machine learning—are either unaware of these tools or find them difficult to use due to the steep learning curve of processing raw sequences. This is compounded by the fact that public datasets often lack phylogenetic trees, providing only abundance tables and taxonomic classifications. To address this, we present a comprehensive review of phylogenetic tree construction techniques for microbiome data (16S rRNA or whole-genome shotgun sequencing). We outline the strengths and limitations of current methods, offering expert insights and step-by-step guidance to make these tools more accessible and widely applicable in quantitative microbiome data analysis.

## 1 Introduction

Advances in microbiome research have increasingly demonstrated that the human microbiome— the community of microorganisms residing in and on our bodies—plays a crucial role in maintaining health and influencing disease. For instance, evidence from various studies suggests that microbial dysbiosis plays a critical role in conditions such as obesity [1], [2], cardiovascular diseases [3], [4], and other disorders [5], [6]. To facilitate studies examining associations between microbial compositions and patient outcomes, researchers use high-throughput next-generation sequencing (NGS) [7]. This technology enables the quantification and analysis of the collective genomic content in biological samples, allowing for the reconstruction of read counts for different bacterial types to represent the microbial compositions within a community accurately.

**Fig. 1:**
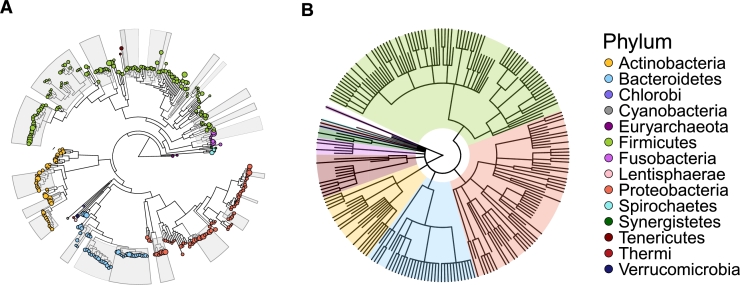
**Cladograms display differences between phylogenetic tree and taxonomy tree.** (A) The phylogenetic tree illustrates not only hierarchical structure but also biological similarities between taxonomy with different lengths of branches. (B) The taxonomy tree only shows the hierarchical structure of taxonomy with the same lengths of branches.

Once NGS data are obtained, quality control, denoising, and alignment steps lead to the construction of phylogenetic trees, which provide a framework to study the evolutionary relationships among microbial species or strains [8]. Unlike traditional taxonomic trees that only depict the hierarchical classification of taxa, phylogenetic trees represent the evolutionary history and genetic similarities between organisms [9]. This deeper understanding of evolutionary relationships is critical for interpreting the compositional and functional dynamics of microbial communities. As shown in Fig. 1, phylogenetic trees not only show evolutionary relationships between microorganisms but also illustrate lineage and sequence similarities according to the length of branches [9]. We aligned the two cladograms to the same angle to maintain consistency in the representation of phylum taxonomies across both trees. While they share similar taxonomic assignments, their tree structures differ significantly.

**Fig. 2:**
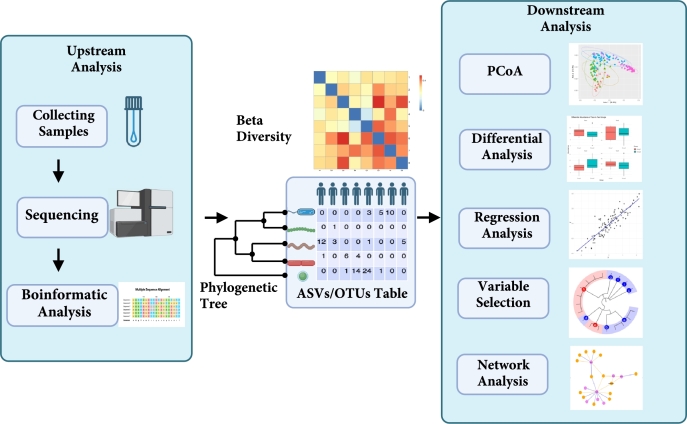
**General workflow for microbiome analysis focused on phylogenetic tree construction for 16S rRNA data.** The upstream analysis begins with sample collection and sequencing, followed by bioinformatic processes such as quality control, denoising, and sequence alignment. Once the feature table is obtained, beta diversity can be calculated by calculating the dissimilarities between samples, and phylogenetic trees can be constructed by calculating the similarities between taxa. For downstream analysis, various options are available, including Principal Coordinate Analysis (PCoA), differential analysis, regression analysis, variable selection, and network analysis, among others.

Phylogenetic trees play a pivotal role in microbiome analysis, linking upstream and downstream analyses (as shown in Fig. 2). As an output of upstream processes, they are generated from raw sequencing data to represent the evolutionary relationships among microbial taxa. These trees then become essential inputs for downstream steps, enabling further statistical and functional exploration of microbial communities. To clarify these stages, we define upstream and downstream analyses as follows. Upstream analysis refers to the processing of raw sequencing data and the generation of quantitative measurements (e.g., abundance tables, taxonomic assignments, and phylogenetic trees) that profile microbial composition. Downstream analysis utilizes these outputs to perform tasks such as diversity analysis, statistical modeling, association studies with clinical or environmental outcomes, and creating informative data visualizations. [10].

For upstream researchers, phylogenetic trees have broad applications in biology, including phylogenetic placement of metagenomic reads, taxonomic affiliation, understanding evolutionary history, and classifying genes into families [11], [12], [13], [14]. For downstream researchers, phylogenetic trees are crucial for quantitative microbiome data analysis [15], [16]. Incorporating phylogenetic information into these analyses enhances understanding and interpretation of microbial communities [17], [18], [19], [20]. As illustrated in Fig. 2, many classical statistical methods utilize phylogenetic trees, such as Principal Coordinate Analysis (PCoA) [21], differential abundance testing [22], regression analysis, variable selection [17], and network analysis [23]. These methods aid analysis of complex microbial data sets, advancing knowledge of microbial communities [24], [25]. A significant example is one of the widely used beta diversity measures, UniFrac dissimilarity [26], which leverages phylogenetic trees to obtain a measure of closeness for related species and construct a non-Euclidean distance that accurately reflects the differences between samples.

However, many researchers face challenges in obtaining phylogenetic trees using microbiome sequencing data because public databases often do not provide preconstructed phylogenetic tree files. While data repositories such as Qiita [27], MG-RAST [28], and the National Center for Biotechnology Information (NCBI) Sequence Read Archive (SRA) [29] offer raw sequencing data for both 16S rRNA and whole genome shotgun (WGS) sequencing, they rarely include the associated phylogenetic trees. Although the pipeline for constructing phylogenetic trees from 16S rRNA sequencing data is relatively well established, the process for WGS sequencing data is more complex and less standardized, requiring advanced tools and a deeper understanding of the data. Moreover, researchers without a bioinformatics background often face a steep learning curve in navigating the bioinformatics pipelines necessary for phylogenetic tree construction.

In this review, we aim to bridge the gap between upstream and downstream researchers by providing a comprehensive overview of the tools and methods used to construct phylogenetic trees from both 16S rRNA and WGS data. We introduce and compare various tools and approaches used to make the upstream process of tree construction more accessible to biostatisticians and other downstream researchers. Our goal is to offer quick and practical guidance to help researchers build phylogenetic trees efficiently, allowing them to focus on the downstream statistical analyses that rely on essential methods illustrated in Fig. 2.

## 2 Microbial sequence alignment and phylogeny

For both 16S rRNA and WGS data, the core steps involve in constructing a phylogenetic tree are generally similar, encompassing sample collection, quality control and denoising, sequence alignment, and tree construction. However, the key differences between these two sequencing methods lie in the sequence alignment and tree construction stages.

Sequence alignment and phylogenetic tree construction are closely related but serve distinct roles in bioinformatics. A phylogenetic tree is typically a byproduct of sequence alignment, where the tree represents evolutionary relationships based on the similarities between aligned sequences. The process of alignment lays the foundation for the tree, allowing for the comparison of homologous sequences by positioning them to reflect evolutionary events such as mutations or conserved regions. However, sequence alignment is far more versatile than just being a precursor to tree construction, as it also serves various other purposes in microbial genomics. For instance, sequenced RNA, such as expressed sequence tags and full-length mRNAs, can be aligned to a sequenced genome to identify gene locations and gain insights into alternative splicing [30] and RNA editing [31]. Sequence alignment is also fundamental to genome assembly, where overlapping sequences are aligned to form contigs (long stretches corresponding to contiguous regions, [32]). Phylogenetic tree construction is only one of the many applications of aligned sequences, reinforcing that alignment serves as a versatile tool beyond evolutionary studies.

When comparing the use of sequence alignment in 16S rRNA and WGS data, key differences emerge. For 16S rRNA data, sequence alignment and phylogenetic tree construction rely primarily on sequence similarities, as 16S rRNA sequences focus on conserved regions of the genome, making direct comparisons of sequences easy and informative. In contrast, for WGS data, both sequence alignment and tree construction depend on reference databases owing to the increased complexity of WGS sequences. The WGS sequences capture the entire genome, including not only informative (coding) regions but also noninformative (non-coding) regions, requiring more sophisticated methods of sequence alignment [33].

### 2.1 Sequence alignment

Sequence alignment is the process of arranging sequences of DNA, RNA, or proteins [34], [35], [36] to identify regions of similarity that may indicate functional, structural, or evolutionary relationships among the sequences [37]. A detailed description of alignment procedures for different data types is provided in Section 1 of the supplementary materials. Through sequence alignment, gaps are inserted into sequences to optimize the match between similar regions, helping to reveal evolutionary patterns [34]. By aligning sequences, researchers can systematically compare bases or amino acids at corresponding positions in DNA, RNA, or protein sequences. This process helps in detecting conserved regions, mutations, or variations among sequences, which are critical for understanding evolutionary relationships, gene function, and structural similarities. These aligned regions are then used to construct phylogenetic trees, revealing the evolutionary distance and relationships between species or samples.

The two primary types of sequence alignment are global alignment and local alignment. Global alignment compares two sequences by aligning their entire lengths to maximize overall similarity; the Needleman-Wunsch algorithm [34] is a commonly used method for this. Local alignment, on the other hand, focuses on sequence regions with the highest density of matches, typically using the Smith-Waterman algorithm [38]. Specifically, when applying sequence alignment to long reads, several challenges arise. Global alignment, while offering a more comprehensive comparison across the entire length of sequences than local alignment does, requires significantly more computational resources, particularly when dealing with long or complex reads. Local alignment is better suited for long reads as it focuses on aligning regions of high similarity. This reduces the computational burden, making local alignment faster and more efficient for WGS applications. However, local alignment may sacrifice accuracy, especially when applied to reads with noninformative regions or when crucial sequence variations lie outside the aligned regions.

Both global and local alignment can be applied to 16S rRNA and WGS data. However, because of the respective advantages and disadvantages of these data types, global alignment is more commonly used for 16S rRNA data. Tools like MAFFT [39], [40], Clustal Omega [41], and MUSCLE [36] are popular choices for 16S data, as their sequences are relatively short and focus on highly conserved regions, making global alignment ideal for maximizing sequence similarity across the entire sequence length. In cases where efficiency is a priority, local alignment methods such as Lambda [42] also can be used.

For WGS data, which is more complex than 16S data and often involves much longer sequences, local alignment methods like Bowtie 2 [43], HISAT 2 [44], and Minimap 2 [45] are generally preferred. Local alignment is more efficient than global alignment for WGS because it focuses on aligning only the most similar regions in sequences, which is critical given the vast amount of noninformative or repetitive genomic content in whole genomes.

Another key difference between 16S and WGS sequence alignment is the need for a reference database. For WGS, a reference database is essential owing to the complexity of the data. WGS sequences span entire genomes, including both coding and noncoding regions, as well as potentially mobile genetic elements. Without a reference database to map these diverse, extensive regions, accurately assigning taxonomy and performing phylogenetic analysis would be challenging. In contrast, 16S rRNA sequencing targets a well-conserved gene, enabling direct alignment of sequences without always requiring such comprehensive reference databases.

### 2.2 Tree construction methods

Another crucial difference between 16S rRNA and WGS data lies in the strategies used to build phylogenetic trees after sequence alignment. For 16S rRNA data, tree construction is typically based on calculating sequence similarities between the conserved regions of the 16S gene. This allows for the application of various mathematical algorithms, such as Neighbor-Joining (NJ) [46], maximum likelihood [47], and Bayesian inference [48], [49]. Researchers have developed many tools for tree construction based on these theories, including FastTree [50], RAxML [51], IQ-TREE [52], and PhyML [53] for maximum likelihood methods, and BEAST [54], PhyloBayes [55], and MrBayes [56] for Bayesian inference. In Section 2 of supplementary materials, we provide a detailed description of different phylogenetic tree construction methods.

Building phylogenetic trees from 16S data is generally fast owing to the relatively short and conserved nature of the sequences. However, this can result in misplacement of highly similar sequences belonging to different biological groups, leading to inaccuracies in phylogenetic tree construction [57].

In contrast, WGS data presents a more complex challenge owing to the high variability across different genome regions. Directly building phylogenetic trees from sequence similarities alone for WGS data is difficult. A common approach is to create a subset of a reference phylogenetic tree. A reference tree is a preconstructed phylogenetic tree containing comprehensive evolutionary information from a large database that enhances the reliability of the results of phylogenetic tree construction. The reference tree built from WGS data provides a more accurate framework for identifying and correcting potential misplacements or errors in phylogenetic trees constructed from 16S rRNA data. This is because the 16S tree relies primarily on sequence similarities and is often generated using tools like FastTree, which may limit its precision. In contrast, WGS-based phylogenetic trees incorporate more comprehensive genomic information, leading to greater accuracy and consistency in the classification of taxa. Additionally, a reference tree reduces computational costs for downstream researchers when phylogenetic trees are required for further analysis.

In summary, 16S rRNA phylogenetic trees are often built on the basis of sequence similarities, whereas WGS relies on reference trees. In the discussion section, we will address potential improvements in constructing 16S phylogenetic trees to overcome these limitations.

## 3 Tools for phylogenetic tree construction using 16S rRNA and WGS data

**Fig. 3:**
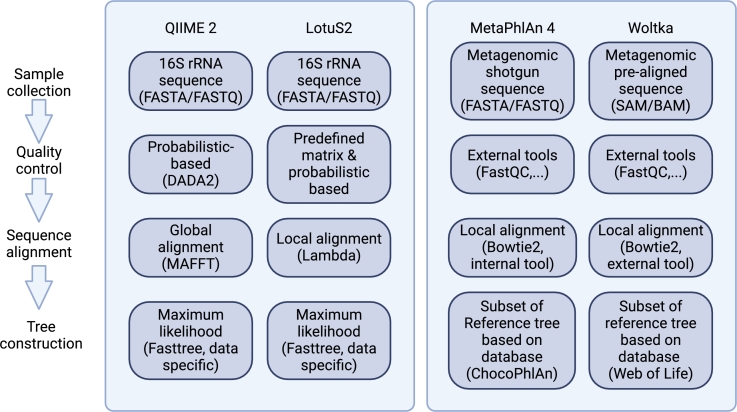
Framework for different tools used in microbiome analysis. There are two main sequencing methods for microbiome studies: 16S rRNA sequencing and shotgun sequencing. For 16S rRNA sequencing, QIIME 2 and LotuS2 are included in the manuscript. For shotgun sequencing, tools such as MetaPhlAn 4 and Woltka are widely used. The differences between different steps are illustrated in each box for each tool.

In this section, we explore the tools and workflows used for phylogenetic tree construction 16S rRNA and WGS data. Researchers have developed different pipelines to address the unique challenges of both sequencing methods. Specifically, we describe below some of the most widely used tools for both 16S and WGS data, such as QIIME 2 [58] and LotuS2 [59] for 16S rRNA data, and MetaPhlAn 4 [60] and Woltka [61] for WGS data. In Fig. 3, we present four tools discussed in this paper to explicitly highlight the differences in the features of these four tools.

### 3.1 Tools for 16S rRNA phylogenetic tree construction

While the core steps for building phylogenetic trees, as outlined in section 2, are similar for both 16S rRNA and WGS data, there are significant differences in how these steps are executed for each data type. Specifically, in 16S rRNA analysis, Operational Taxonomic Units (OTUs) [62] and Amplicon Sequence Variants (ASVs) play crucial roles in linking the denoising and clustering of sequences with the processes of sequence alignment and phylogenetic tree construction.

OTUs and ASVs are key to organizing raw 16S sequences into biologically meaningful units. OTUs are generated by clustering sequences based on similarity thresholds (typically 97%), which groups closely related sequences as proxies for species [63]. Although this has been widely used, the arbitrary clustering threshold can sometimes obscure fine taxonomic distinctions. ASVs [64], generated through denoising algorithms like DADA2 [65], offer higher resolution than OTUs by distinguishing sequences at 100% similarity. Unlike OTUs, which cluster similar sequences, ASVs retain even small sequence differences, allowing for more detailed analysis of microbial communities.

### 3.2 Introduction to QIIME 2 and LotuS2

QIIME 2 and LotuS2 are both comprehensive tools for analyzing 16S rRNA data, but they exhibit key similarities and differences in their workflows. From a global perspective, QIIME 2 provides a fully integrated platform for microbiome data analysis, where each core step is built into the internal system of QIIME 2, allowing for the customization of parameters at every stage. This integration makes QIIME 2 well suited for users who have to adjust parameters to accommodate specific data characteristics. However, the level of customization also introduces a steep learning curve, which can be challenging for downstream researchers new to bioinformatics. In contrast, LotuS2 provides a streamlined approach with which users only have to input raw sequence files and select tools with default parameters. The platform automatically produces all upstream outputs, including an ASV/OTU table, taxonomic classification, and phylogenetic tree, making it particularly appealing for high-throughput environments. This approach is very user-friendly but is less flexible than QIIME 2, limiting users' ability to customize specific steps in the upstream process.

To comprehensively compare QIIME 2 and LotuS2, we examined each core step involved in the phylogenetic tree construction pipeline. Both tools use raw sequence files in FASTA or FASTQ format as input, ensuring compatibility with a wide range of sequencing outputs.

Regarding quality control, QIIME 2 employs DADA2, a denoising algorithm that models sequence abundance using a Poisson model based on quality scores obtained during sequencing [65]. This probabilistic approach enables DADA2 to handle low-quality sequences effectively, distinguishing between biological variants and sequencing noise. By not discarding all low-quality sequences, DADA2 captures valuable information from noisy data, which enhances the accuracy of downstream analysis. In comparison, LotuS2 adopts a more stringent quality control strategy, relying on a combination of predefined quality control metrics, including average quality scores, detection of homonucleotide repeats, removal of reads lacking amplicon primers, and a probabilistic model [59], while this approach is highly efficient at filtering high-quality reads, it may be overly strict, potentially excluding informative sequences from lower quality datasets.

Sequence alignment is another area in which these two tools differ significantly. QIIME 2 commonly uses MAFFT for alignment. MAFFT is a tool designed for global alignment that excels in aligning entire sequences, prioritizing accuracy but requiring more computational resources than local alignment methods [40]. Conversely, LotuS2 uses Lambda, a local alignment method that offers increased speed but sacrifices some accuracy, especially when important differences across the full lengths of sequences are present. As discussed in section 2.1, these strategic differences between global and local alignment reflect the trade-offs between precision and computational efficiency.

For phylogenetic tree construction, both QIIME 2 and LotuS2 use FastTree, a tool that employs the maximum likelihood method to build trees. FastTree constructs phylogenetic trees based solely on sequence similarities and does not rely on a reference database. It is highly efficient and capable of handling large datasets, but it is slightly less accurate than more computationally intensive methods [50], [66]. While this makes FastTree a suitable choice for most routine 16S rRNA sequence analyses, it may not be ideal for cases where maximum accuracy is critical.

In terms of output files, both QIIME 2 and LotuS2 generate ASV/OTU tables, taxonomy tables, and phylogenetic trees. Notably, LotuS2 can integrate these outputs into a “phyloseq” object [67], providing added convenience for downstream researchers conducting further statistical analysis. In Section 3 of the supplementary materials, we have applied QIIME 2 and LotuS2 to a real data example to show the difference of phylogenetic tree structures from the perspective of taxonomy distribution. Table 1 in supplementary materials provides a summary of comparisons for QIIME 2 and LotuS2 for each core step.

**Table 1:** Literature summary.

**Topic**	**Method**	**Description**	**Tools**	**References**
Alignment	DNA Alignment	Aligning DNA sequences through pairwise sequence alignment	Bowtie 2, HISAT 2, Minimap 2, MAFFT	[38], [43], [44], [45], [40]
	Protein Alignment	Aligning amino acid sequences to identify regions of similarity	Clustal Omega, MAFFT	[41], [40]

Phylogenetic tree construction	Distance-based method	Mapping a dissimilarity matrix representing biological data to a tree structure	Neighbor-joining (NJ), Unweighted pair group method with arithmetic mean (UPGMA), Molecular Evolutionary Genetic Analysis (MEGA), Tree analysis using New Technology (TNT)	[68], [69], [70], [71]
	Maximum likelihood method	Identifying tree that maximizes the likelihood of observing the given sequence data under a specific evolutionary model	FastTree, RAxML, IQ-Tree, PhyML	[66], [51], [52], [53]
	Bayesian inference method	Combining the prior information of parameters with the likelihood of sequence data to obtain posterior information of parameters	BEAST, PhyloBayes, MrBayes	[48], [49], [54], [55], [56]

Phylogenetic tree construction in microbiome	16S Sequencing method	Amplifying and sequencing the 16S rRNA gene to identify and classify bacteria, followed by alignment and tree-building methods to elucidate evolutionary relationships.	QIIME 2, MAFFT, FastTree	[58], [40], [39], [50]
	Shotgun Metagenomic Sequencing method	Sequencing random fragments of microbial genomes, followed by assembly, annotation, and alignment to reconstruct evolutionary relationships across the entire microbial community.	MetaPhlAn 4, Woltka, Bowtie 2	[72], [61], [73], [43]
	whole genome shotgun sequencing method	Sequencing random fragments of an entire genome, providing comprehensive coverage of all genetic material, including coding and non-coding regions, mobile genetic elements, and strain-level variations.	Kraken 2, Bowtie 2	[74], [43]

Although LotuS2 provides an automated, streamlined workflow, it still requires users to study tutorials when selecting configuration files for quality control. Since LotuS2 uses predefined metrics for quality control, it offers various configuration files with different parameters. Without familiarity with these configurations, users may encounter time-consuming debugging processes. Additionally, the reliance on predefined metrics makes LotuS2 less flexible and customizable compared to QIIME 2. Therefore, QIIME 2 is more widely recognized for its comprehensive capabilities within the microbial research community. As an open-source platform, QIIME 2 not only supports reproducible research but also fosters innovation by enabling the global research community to contribute new plugins, workflows, and features for microbiome data analysis. This adaptability enables researchers to integrate the latest tools and methodologies into their analyses. Therefore, although LotuS2 simplifies upstream analysis with predefined options, we recommend QIIME 2 as the primary tool for 16S rRNA phylogenetic analysis given its flexibility, broad community support, and ability to incorporate cutting-edge advancements.

### 3.3 Guide for phylogenetic tree construction using QIIME 2

Phylogenetic trees are built with QIIME 2 in four steps:

Step 1: Import raw FASTA/FASTQ files and demultiplex them.
Step 2: Denoise sequences and generate an ASV table.
Step 3: Align sequences using the MAFFT plugin.
Step 4: Build a phylogenetic tree using the Fasttree plugin.

For the detailed guidance of programming, please refer to the website https://raytaoliu.github.io/phylogeny/posts/phylogenetic_trees/.

### 3.4 Tools for WGS phylogenetic tree construction

Similar to the use of OTUs and ASVs in 16S rRNA data analysis, WGS data analysis also involves clustering sequences into biologically meaningful units. However, because of the vast diversity and amount of WGS data, researchers have developed more advanced concepts for WGS data analysis, such as metagenomic OTUs (mOTUs) [75], Metagenome-Assembled Genomes (MAGs) [60], Species-Level Genome Bins (SGBs) [76], and Operational Genome Units (OGUs) [61].

Metagenome-Assembled Genomes (MAGs) became a breakthrough concept in 2013, allowing researchers to assemble near-complete microbial genomes directly from metagenomic data without the need for cultivation [77], [78]. MAGs are constructed by binning sequences from metagenomic datasets based on sequence similarity, coverage patterns, and other genomic features, followed by assembly into longer contiguous sequences (contigs) and scaffolds. This approach revolutionized the field by recovering genomes from unculturable species, shedding light on microbial diversity, and facilitating the discovery of novel species. Today, MAGs play a foundational role in many upstream analyses, offering high-resolution, species-level insights essential for microbial ecology, evolutionary studies, and comparative genomics.

Metagenomic Operational Taxonomic Units (mOTUs), introduced in 2013, offer a faster, more efficient way to cluster WGS data compared to full genome assembly [79]. By using species-specific marker genes instead of requiring full genomes, mOTUs significantly reduce the computational burden [80]. This makes them particularly useful when working with fragmented genomes, allowing for rapid and accurate taxonomic profiling even in incomplete datasets. The flexibility and efficiency of mOTUs have made them popular in large-scale metagenomic studies, especially when profiling both known and unknown species.

Species-Level Genome Bins (SGBs), introduced around 2019, build upon MAGs by grouping genomes based on species-level similarity [81]. SGBs combine known reference genomes with novel MAGs, creating a comprehensive framework that expands our understanding of microbial diversity. By clustering previously uncharacterized genomes at the species level, SGBs provide a powerful tool for classifying novel species and integrating them into established taxonomies, significantly enhancing our knowledge of the microbial world [72].

Operational Genome Units (OGUs), a more recent development from 2022, take genome classification to the next level by using whole-genome sequences instead of marker genes [61]. This allows for genome-wide variation analysis, providing a more refined view of microbial relationships. OGUs are particularly valuable for high-resolution phylogenetic studies, offering detailed evolutionary insights across entire genomes and enabling researchers to trace microbial evolution with unprecedented precision.

In summary, OTUs to mOTUs, MAGs, OGUs, and SGBs reflect the advancement of microbiome research from basic taxonomic clustering to genome-wide approaches that offer more detailed insights into microbial diversity, evolution, and function. Each concept builds upon its predecessors, addressing their limitations and pushing the field toward more accurate, comprehensive, and functional analyses of microbial communities. This trajectory underscores the increasing importance of whole-genome data in microbiome research and the ongoing refinement of tools and methods to handle the complexity of microbial ecosystems.

### 3.5 Introduction to MetaPhlAn 4 and Woltka

In this section, we compare the four core phylogenetic tree construction steps using MetaPhlAn 4 and Woltka as described in section 2. A key distinction between constructing phylogenetic trees from 16S rRNA and WGS data lies in the use of reference databases for sequence alignment and phylogenetic tree construction. Therefore, we also examine the reference databases used with MetaPhlAn 4 and Woltka, exploring how both tools build and use databases to perform taxonomic classification and phylogenetic analysis.

From a global perspective, MetaPhlAn 4 operates as an end-to-end pipeline, directly processing raw sequence files (FASTA/FASTQ) and generating taxonomic profiling outputs. This seamless integration streamlines the workflow in sequence alignment and taxonomy classification, optimizing processing speed and efficiency and making MetaPhlAn 4 well suited for high-throughput environments. In comparison, Woltka primarily functions as a taxonomy classification tool that relies on alignment files (SAM/BAM) as inputs. This introduces additional complexity, as users must manually perform sequence alignment using external tools like Bowtie 2 before running Woltka. This extra step can result in a steeper learning curve, particularly for downstream researchers who are less familiar with alignment processes than others.

For details of each step of the phylogenetic tree construction, MetaPhlAn 4 accepts raw sequence data in FASTA/FASTQ format and processes it directly, streamlining the workflow. In contrast, Woltka requires prealigned sequence data in SAM/BAM format, which adds an additional preprocessing step.

Quality control is essential when working with WGS data in both MetaPhlAn 4 and Woltka. Ensuring that only high-quality reads are retained is critical for accurate taxonomic and functional profiling, as it directly impacts the reliability of downstream analyses. However, in contrast with tools like QIIME 2 and LotuS2, which integrate quality control tools such as DADA2, MetaPhlAn 4 and Woltka do not include internal quality control mechanisms. Instead, they rely on external tools like FastQC [82], which provides a straightforward way to perform quality checks of raw sequence data from high-throughput sequencing pipelines.

In terms of sequence alignment, MetaPhlAn 4 automates the process by integrating Bowtie 2 [43] as a plug-in, making it user-friendly and efficient, particularly for high-throughput analyses. This automation reduces manual effort for researchers, while Woltka requires users to manually apply Bowtie2 for alignment before proceeding to taxonomic classification. Although this offers flexibility, it introduces additional complexity and can increase the time and computational resources needed for sequence alignment. When processing sequences with Woltka, this manual sequence alignment step may pose a challenge for users who are less familiar with alignment tools, especially when working with large datasets.

For both 16S and WGS data, a reference database is required for sequence alignment and phylogenetic tree construction. The choice of database can significantly influence the structure and accuracy of the resulting phylogenetic trees. In particular, the database affects which taxa are detected, how the taxa are classified, and how evolutionary relationships are inferred, ultimately shaping the overall structure of the phylogenetic tree [25]. MetaPhlAn 4 uses the ChocoPhlAn database [83], which is built on core marker genes selected for their ability to distinguish between species. This SGBs database includes more than 5.1 million marker genes across 21,978 known SGBs and 4,992 unknown SGBs [72]. This focused approach to identifying species ensures a high-resolution taxonomy assignment for well-represented species, leading to precise phylogenetic trees. However, MetaPhlAn 4 may struggle with novel or rare organisms not represented in the reference database, limiting its ability to detect unknown taxa and affecting the completeness of the tree. In comparison, Woltka uses the Web of Life (WoL) database [73], which is based on a phylogenetic framework of 10,575 bacterial and archaeal genomes. WoL employs 381 highly conserved marker genes to map whole-genome sequences, providing broad coverage of taxa, including many that may not be well-characterized. This allows Woltka to detect more novel or unclassified taxa than MetaPhlAn, resulting in a more comprehensive though less specific phylogenetic tree. The inclusion of unclassified or ambiguous taxa can sometimes introduce uncertainty into a tree structure, inhibiting the clarity of evolutionary relationships among species.

Additionally, for phylogenetic tree construction, both MetaPhlAn 4 and Woltka rely on reference trees for phylogenetic inference. However, the reference databases and the tree-building approaches they employ can significantly impact the construction and resolution of these reference trees, as we illustrate with a real data example in Section 4 of the supplementary material. MetaPhlAn uses PhyloPhlAn [84], which leverages highly conserved marker genes to efficiently build species-level phylogenies. In comparison, Woltka employs ASTRAL [85], which is better suited for detailed strain- or subspecies-level analyses by integrating gene trees and handling incomplete lineage sorting. These distinctions highlight how these tools approach tree building differently depending on the evolutionary scope of the analysis.

For output files of results, both MetaPhlAn and Woltka generate taxonomy profiling tables that describe microbiome composition. A key distinction between them is that MetaPhlAn 4, by default, generates relative abundance tables, whereas Woltka produces absolute abundance tables. Absolute abundance tables are often preferred by downstream researchers for statistical modeling and differential abundance testing because they provide raw data that can be directly integrated into statistical frameworks. Woltka's count tables in particular enhance its versatility for subsequent analyses, including functional profiling and differential abundance analysis.

In summary, while MetaPhlAn's marker gene-based approach offers a fast and efficient solution for many studies, researchers seeking more detailed resolution, raw data for statistical modeling, or greater adaptability in their workflows should consider Woltka. In Table 2 of the supplementary materials, we have provided a summary table to compare each step of phylogenetic tree constructions using MetaPhAln 4 and Woltka.

### 3.6 Guide for phylogenetic tree construction using Woltka

A reference tree for Woltka can be downloaded from https://biocore.github.io/wol/data/trees/. The steps to construct phylogenetic trees using Woltka are as follows:

Step 1: Install a Custom Database with Bowtie 2: install the Web of Life database [73] as the reference database for Bowtie 2.
Step 2: Sequence Alignment: use Bowtie 2 to align raw sequence files against the WoL database.
Step 3: Taxonomy profiling: perform taxonomy classification using Woltka to generate OGU tables.
Step 4: Build the Phylogenetic Tree: use the ape package in R [86], you can create a subset tree by mapping your OGUs to the reference tree and build a phyloseq object [67].

In sections 5 of the supplementary materials, we provide detailed descriptions of phylogenetic tree construction using MetaPhlAn 4. For the detailed guidance of programming, please refer to the website https://raytaoliu.github.io/phylogeny/posts/phylogenetic_trees/.

## 4 Discussion

Phylogenetic tree construction for 16S rRNA data is generally based on sequence similarities of the 16S rRNA gene, focusing on specific marker regions. Although many tools are available to streamline this process, significant challenges remain. A primary concern is that similarity-based methods can sometimes misgroup sequences due to high homology among different species, leading to misclassification and inaccurate evolutionary relationships. A promising future direction could involve building a comprehensive reference tree specifically for 16S rRNA data, similar to the approach used for WGS data. SATé-enabled phylogenetic placement (SEPP) [57] is a method that facilitates the insertion of 16S rRNA sequences into pre-existing phylogenetic trees, allowing researchers to build and extend 16S reference trees efficiently. However, these reference trees are often less accurate compared to those constructed using WGS data, which leverages entire gene sequences to provide finer resolution and better capture evolutionary relationships for taxa. Additionally, SEPP currently relies on outdated versions of 16S rRNA databases, such as older versions of SILVA and Greengenes [87] databases, limiting the precision of phylogenetic placement. Moving forward, developing a consensus, up-to-date reference tree for 16S rRNA data will be essential to improve the accuracy and reliability of these analyses.

For WGS data, tree construction typically utilizes reference trees derived from whole-genome comparisons within comprehensive genomic databases, offering a broader, genome-wide evolutionary context. Tools like MetaPhlAn 4 and Woltka, which were introduced earlier in Section 3.5, facilitate this process by leveraging these databases for accurate phylogenetic placement and analysis. In contrast, alternative tools such as Kraken 2 [74], introduced in Section 6 of the supplementary materials, take a different approach by focusing on rapid classification through k-mer-based methods, rather than leveraging whole-genome reference trees for phylogenetic analysis. However, WGS data presents distinct challenges due to the complexity of entire genomes, which encompass both coding and noncoding regions. The growing number of tools for quality control, taxonomic classification, sequence alignment, and phylogenetic tree construction requires a careful evaluation of each step in the analysis pipeline. For instance, FastANI [88] calculates the average nucleotide identity (ANI) between two genomes by fragmenting the query genome and comparing it to a reference genome, making it particularly valuable for working with MAGs and SGBs, where precise genome assembly is essential. Importantly, after calculating the ANI scores between multiple genomes, the resulting similarity matrix can be used as input for phylogenetic tree construction, thereby providing an evolutionary context for the relationships between genomes. As reference databases continue to expand, the demand for efficient tree construction tools that can seamlessly incorporate updates from these genomic resources is increasing.

Another challenge arises when researchers obtain different upstream results with the same dataset across different platforms. This lack of standardization presents a significant barrier to generating reproducible and comparable microbiome analysis results for both 16S rRNA and WGS data. Without a standardized reference database and reference tree, taxonomic classifications and phylogenetic tree constructions can vary significantly depending on the platform or tool used. The variation in reference databases and trees affects how microbial sequences are aligned, classified, and interpreted, leading to inconsistencies in the number of taxa identified and their evolutionary relationships. Such discrepancies hinder effective data comparison across studies and make integration of 16S rRNA and WGS sequencing results challenging. Developing a consensus, regularly updated reference database and reference tree would resolve this issue. Such a resource would ensure consistency in taxonomic identification across platforms, facilitating reproducible and comparable results. An integration database for 16S rRNA and WGS data called Greengenes 2 [89] has made strides in this direction by integrating both 16S and WGS data into a comprehensive reference tree. However, a more streamlined, universally accepted solution is still needed to bridge the gap between ASVs and genome-level identifiers, ultimately enabling more reliable and reproducible microbial community analyses. As for downstream researchers, the reliability and consistency of phylogenetic trees are essential for generating consensus results for the same study, especially when developing novel statistical models that integrate phylogenetic information. As more advanced statistical models incorporate phylogenetic trees [90], [91], [23], the need for a combined, consensus reference tree for both 16S and WGS data becomes even more pressing. This would result in more comparable results of different statistical models and improve the accuracy of microbial community analysis.

Moreover, the development of computer hardware and artificial intelligence (AI) is playing an increasingly important role in microbial research [92], [93], leading to improvements in the efficiency and accuracy of microbial data processing. There are already many existing methods using deep learning in upstream analysis including phylogenetic analysis [94], [95], [96], [97]. As tools for building phylogenetic trees become more advanced and user-friendly, they will enable downstream researchers to develop novel statistical models that can provide deeper insights into microbial communities. However, despite the progress in upstream applications, there remains a notable gap in developing innovative statistical models that fully integrate AI and microbiome data, with only a few researchers currently exploring this area [98]. The combination of AI-driven methods and statistical modeling hold significant potential for both upstream and downstream researchers, opening up new opportunities to unravel the complexities of microbial ecosystems.

## Code availability

The code for tree visualization used in the main manuscript and supplementary material is available in https://github.com/bioscinema/metaphylogeny. The detailed tutorial for constructing phylogenetic trees with different tools is available in https://raytaoliu.github.io/phylogeny/posts/phylogenetic_trees/.

## CRediT authorship contribution statement

**Ruitao Liu:** Writing – review & editing, Writing – original draft, Visualization, Investigation, Conceptualization. **Xi Qiao:** Writing – review & editing. **Yushu Shi:** Writing – review & editing. **Christine B. Peterson:** Writing – review & editing. **William S. Bush:** Writing – review & editing. **Fabio Cominelli:** Writing – review & editing, Funding acquisition. **Ming Wang:** Writing – review & editing. **Liangliang Zhang:** Writing – review & editing, Visualization, Supervision, Resources, Project administration, Methodology, Funding acquisition, Conceptualization.

## Declaration of Competing Interest

The authors declare that they have no known competing financial interests or personal relationships that could have appeared to influence the work reported in this paper.

## Data Availability

The processed data used in supplementary material is available in https://github.com/bioscinema/metaphylogeny.The raw sequencing data used in supplementary materials is available in https://qiita.ucsd.edu/study/description/11808.

## Appendix A Supplementary material

MMC The supplementary material consists of six sections. Sections 1 and 2 provide detailed descriptions of sequence alignment methods and phylogenetic tree construction algorithms. Sections 3 and 4 use real data examples to demonstrate differences in phylogenetic tree construction for 16S rRNA and WGS data, respectively. Section 5 offers comprehensive guidance on using MetaPhlAn 4 for constructing phylogenetic trees with WGS data. The final section introduces Kraken 2. MMC
