# Supplementary material for: Constructing phylogenetic trees for microbiome data analysis: A mini-review
Source: Comput Struct Biotechnol J. 2024 Oct 24;23:3859–68. doi: 10.1016/j.csbj.2024.10.032 (PMC11564040; doi:10.1016/j.csbj.2024.10.032)
Supplement: MMC — The supplementary material consists of six sections. Sections 1 and 2 provide detailed descriptions of sequence alignment methods and phylogenetic tree construction algorithms. Sections 3 and 4 use real data examples to demonstrate differences in phylogenetic tree construction for 16S rRNA and WGS data, respectively. Section 5 offers comprehensive guidance on using MetaPhlAn 4 for constructing phylogenetic trees with WGS data. The final section introduces Kraken 2. [file mmc1.pdf]

Supplementary Material: Constructing phylogenetic trees for microbiome data analysis: A mini-review

Ruitao Liu, Xi Qiao, Yushu Shi, Christine B. Peterson, William S. Bush, Fabio Cominelli, Ming Wang, Liangliang Zhang*

October 20, 2024

1 Sequence Alignment

Sequence alignment is an active research area in the field of bioinformatics. It is also a crucial task for phylogenetic tree construction. Sequence alignment is usually one of the most significant steps in a bioinformatic pipeline before phylogenetic tree construction. With the advent of Next Generation Sequencing (NGS) [1] techniques, the throughput of sequence production increases many folds and reduces costs by orders of magnitude. During the process of aligning two or multiple sequences of DNA, RNA, or proteins, sequences are arranged to detect the maximum similarity between these sequences [2]. From a techniques perspective, alignment methods can be divided into pairwise sequence alignment (PSA) and multiple sequence alignment (MSA) [3]. Both PSA and MSA can be applied to various types of data.

1.0.1 DNA alignment

DNA alignment involves aligning two DNA sequences through pairwise sequence alignment, and methods are chosen based on the study's aim. There are three main approaches: global, local, and genomic alignment methods. Global alignment compares two sequences by aligning their entire lengths to maximize overall similarity; the Needleman-Wunsch algorithm [4] is a commonly used method for this. Local alignment, on the other hand, focuses on regions with the highest density of matches, typically using the Smith-Waterman algorithm [5]. Genomic alignment involves aligning short genes to a reference genome, with featured tools including Bowtie 2 [6], HISAT 2 [7], and Minimap 2 [8].

Multiple sequence alignment (MSA) aims to align three or more sequences to extract homology and evolutionary relationships, assuming a common ancestor [9, 10]. Several tools are available for MSA, including Clustal Omega [11], the latest version of the Clustal alignment tool, designed for aligning large datasets. MAFFT is used for aligning medium to large datasets and utilizes the fast Fourier transform for rapid detection of homologous regions [12].

*Corresponding author: Liangliang Zhang
email: lxz716@case.edu
Website:https://cwru-cinema.com/author/lianglianglyon-zhang/

1.0.2 Protein alignment

The principle of alignment for DNA and protein alignment is almost identical but the main difference is the size of matrices applied for protein alignment. BLOSIM (Block Substitution Matrix) [13] and PAM (Point Accepted Mutation) [14] are the two main scoring matrices for protein alignment. Basically, scoring matrices are used to quantify the similarity between amino acid sequences by assigning scores to each possible substitution, thus aiding in the identification of conserved regions and evolutionary relationships [15]. Some tools for DNA alignment can be also used for protein alignment like Clustal Omega [11] and MAFFT [12].

Although both DNA and protein alignments are used to study evolution, protein sequences offer distinct advantages for identifying genes and their evolutionary relationships. DNA sequences are often preferred for detecting evolutionary relationships among closely related individuals due to the higher information content provided by advances in DNA sequencing technologies. However, when it comes to more divergent entities, protein sequences are preferred because they are more conserved and can reveal deeper evolutionary connections. Significant progress in protein sequencing has made it possible to use protein sequences effectively, except when missing values in the sequences undermine their evolutionary information [16].

2 Phylogenetic tree construction methods

Phylogenetic trees can be constructed using two main methods: distance-based and character-based [17]. Distance-based methods infer the tree structure by calculating the genetic distance between pairs of sequences. This distance typically represents the number of differences or evolutionary changes between sequences. The most common distance-based methods are the unweighted pair group method with arithmetic mean (UPGMA) [18] and Neighbor-Joining (NJ) [19]. Both methods rely on the initial creation of a distance matrix. Distance-based methods are known for their speed and ease of understanding. However, they summarize sequence information into a single distance value, which may lead to the loss of detailed evolutionary information. Character-based methods such as Maximum likelihood methods and Bayesian inference methods typically generate a large number of hypothetical trees based on an algorithm and then induce an optimal tree according to certain criteria. In these methods, ”characters” refer to specific features of the sequences being analyzed, such as nucleotide or amino acid positions in the alignment. Each character represents a column in a sequence alignment, and the methods evaluate the evolutionary relationships by analyzing the changes or mutations in these characters across the sequences. Character-based methods provide more detailed evolutionary information by evaluating numerous hypothetical trees to find the most optimal one [20].

2.0.1 Distance-based method

Distance-based phylogenetic trees were first introduced by Cavalli-Sforza and Edwards (1967) [21] and Fitch and Margoliash (1967) [22]. This approach is one of the simplest methods for constructing phylogenetic trees. Distance-based algorithms attempt to solve the NP-hard least-squares phylogeny problem by mapping a dissimilarity matrix representing biological data to a tree metric [23]. Representative methods for distance-based

approaches include NJ and UPGMA [24]. These methods work by clustering nodes at each stage and then forming a new node on the tree [25].

The UPGMA is a simple and widely used method for constructing phylogenetic trees. UPGMA assumes a constant rate of evolution and builds clusters by averaging the distances between groups. This method creates a new distance matrix at each step and updates the tree based on these matrices. However, this assumption can lead to inaccuracies if the rate of evolution varies among lineages. NJ operates similarly to UPGMA in that it creates a new distance matrix at each step and builds the tree based on these matrices [26]. The key difference is that NJ does not construct clusters but directly calculates distances to internal nodes. The NJ method is known for its high accuracy and fewer assumptions in constructing phylogenetic trees. It also offers faster computation speed by using a stepwise construction approach rather than searching for the optimal tree [27]. Besides NJ, there are other methods like least-squares and minimum evolution [28], Molecular Evolutionary Genetic Analysis (MEGA) [29] and Tree analysis using New Technology (TNT) [30].

Although distance-based methods, particularly NJ, are very fast and computationally efficient, making them suitable for evolutionary studies in closely related entities, their accuracy can be compromised by high variation among divergent sequences and taxa. This can lead to unreliable phylogenetic relationships between distant sequences. Also, distance-based methods often struggle with the issue of multiple substitutions at the same site. This can lead to an underestimation of the true evolutionary distance between sequences [31].

2.0.2 Maximum likelihood method

One of the main methods for character-based phylogenetic analysis is the maximum likelihood method. This approach was first proposed by Felsenstein in the early 1980s [32] and has become a popular technique for constructing evolutionary trees. The maximum likelihood method aims to identify the tree that maximizes the likelihood of observing the given sequence data under a specific evolutionary model. These models include JC69 [33], K80 [34], TN93 [35], HKY85 [36], and GTR [37]. Because likelihood methods are based on explicit model assumptions, they reduce the probability of systematic errors compared to other methods. Commonly used maximum likelihood methods include FastTree [38], RAxML [39], IQ-TREE [40] and PhyML [41].

The maximum likelihood method offers several advantages. It allows for explicit model assumptions, which can be evaluated and improved, and it provides the capability to study the processes of sequence evolution and test evolutionary hypotheses. However, if the distribution assumptions are violated, the performance of these methods can be significantly affected [42]. In real practice, it is relatively common for assumptions to be violated due to the complexity and variability of evolutionary processes [43]. When assumptions are not met, it can lead to biased or inaccurate phylogenetic trees. Therefore, it is crucial to carefully evaluate and, if necessary, modify the assumptions of the model to better fit the data. Sensitivity analyses and the use of alternative models can help assess the robustness of the inferred phylogenies under different assumptions. Additionally, the method is computationally intensive, requiring the evaluation of the likelihood function for many possible trees and parameter combinations. If the chosen evolutionary model is incorrect, the statistical properties of the method can be weakened, potentially leading to inaccurate phylogenetic inferences.

Bayesian inference for the phylogenetic tree was first proposed in the 1990s [44, 45]. This method is similar to maximum likelihood but is different as Bayesian inference evaluates the probability distribution of trees given the observed data [46]. Here, Markov chain Monte Carlo algorithms [47] calculate the posterior probabilities that are the foundation of all inference. According to Baye's theorem, combining the prior information of parameters with the likelihood of sequence data can obtain posterior information of taxa. Commonly used Bayesian inference methods include BEAST [48], PhyloBayes [49] and MrBayes [50].

Bayesian Inference method is statistically consistent and offers easy interpretation of results. However, the Bayesian Inference method is also computationally demanding. It can handle large datasets, but the attractive feature of being able to estimate the confidence in the trees through posterior probabilities is offset by the high computational cost of these methods.

Table 1: Comparison of Tools for 16S Phylogenetic Tree Construction and Downstream Analysis

	**QIIME 2**	**LotuS 2**
**Input File**	FASTA/FASTQ	FASTA/FASTQ
**Quality Control and Denoising**	DADA2 for denoising based on Poisson distribution	Based on 21 quality filtering metrics (e.g., average quality, homonucleotide repeats, and removal of reads without amplicon primers)
**Sequence Alignment**	MAFFT (Global alignment)	Lambda (Local alignment)
**Tree Construction**	FastTree for phylogenetic tree construction (Efficient, slightly less accurate)	Also uses FastTree
**Taxonomy Assignment**	Uses reference databases (e.g., SILVA, Greengenes) for flexible classification	Built-in tools, fewer database options for taxonomic assignment
**Diversity Analysis**	Extensive alpha and beta diversity metrics (e.g., UniFrac, Bray-Curtis)	Outputs phyloseq objects but offers fewer integrated diversity metrics
**Visualization Tools**	Comprehensive visualizations (trees, bar plots, ordination plots) integrated within pipeline	Limited visual outputs, requires external tools for advanced visualizations
**Ease of Use**	Integrated, highly customizable with extensive community support	Automated, high-throughput but less flexible and customizable

3 Real data application for 16S rRNA data

In this section, we demonstrate how the choice of upstream pipelines significantly impacts the resulting phylogenetic tree by comparing two different methods: QIIME 2 and LotuS2. Both tools were applied to the same 16S rRNA dataset from Qiita [51], specifically study 11808: "Oral Infections, Glucose Intolerance, and Insulin Resistance" [52]. This dataset comprises 181 samples, and our objective was to compare the taxonomic profiling and phylogenetic outputs generated by the two tools.

Table 2: Comparison of MetaPhlAn 4 and Woltka for Core Steps in Phylogenetic Tree Construction

	**MetaPhlAn 4**	**Woltka**
**Input File**	FASTA/FASTQ files	Alignment files (SAM/BAM)
**Quality Control and Denoising**	Requires external QC tools (e.g., Trimmomatic, FASTQC) before alignment	Requires external QC tools (e.g., Trimmomatic, FASTQC) before alignment
**Sequence Alignment**	Automatic alignment with Bowtie 2 integrated into the pipeline, streamlining the workflow	Requires manual alignment using Bowtie 2 or external alignment tools before classification, adding complexity
**Reference Database**	Uses Species-Level Genome Bins (SGBs) database with over 5.1 million marker genes for well-characterized taxa	Uses Web of Life (WoL) database with 381 conserved marker genes, allowing broader taxonomic coverage
**Tree Construction**	Relies on external reference trees built from SGBs for more concise and resolved phylogenies	Uses external reference trees based on WoL, which captures a broader range of taxa, though sometimes more ambiguous
**Output Type**	Relative abundance tables (provides a more concise set of taxa)	Count tables (captures more taxa, including many unclassified taxa)
**Diversity Analysis**	Outputs relative abundance data for downstream analysis	Outputs count data, ideal for direct use in statistical models and differential abundance testing

Since phylogenetic trees are constructed based on the taxa detected by each pipeline, the relationships among these taxa form the fundamental structure of the tree. Therefore, the number of taxa, their distribution across taxonomic ranks, and the overlap between the tools are key factors that shape the overall tree. Table 3 presents the number of taxa detected by QIIME 2 and LotuS2 across different taxonomic levels, with the overlap between the two pipelines also listed. We use the Silva database for both tools, so the primary differences lie in their quality control methods, which result in different taxonomy distributions. Unlike the WGS data analysis, where different databases were used, leading to more divergent results, here the overall distributions are more comparable. As shown in Figures 1 and 2, the main taxa distributions are similar.

Table 3: Comparison of MetaPhlAn 4 and Woltka across Taxonomic Ranks

	**Phylum**	**Class**	**Order**	**Family**	**Genus**
**QIIME 2**	17	30	70	121	207
**LotuS2**	15	22	57	100	189
**Overlap**	14	22	48	78	133

4 Real Data application for WGS data

In this section, we demonstrate how the choice of upstream pipelines significantly impacts the resulting phylogenetic tree by comparing two different methods: MetaPhlAn 4 and

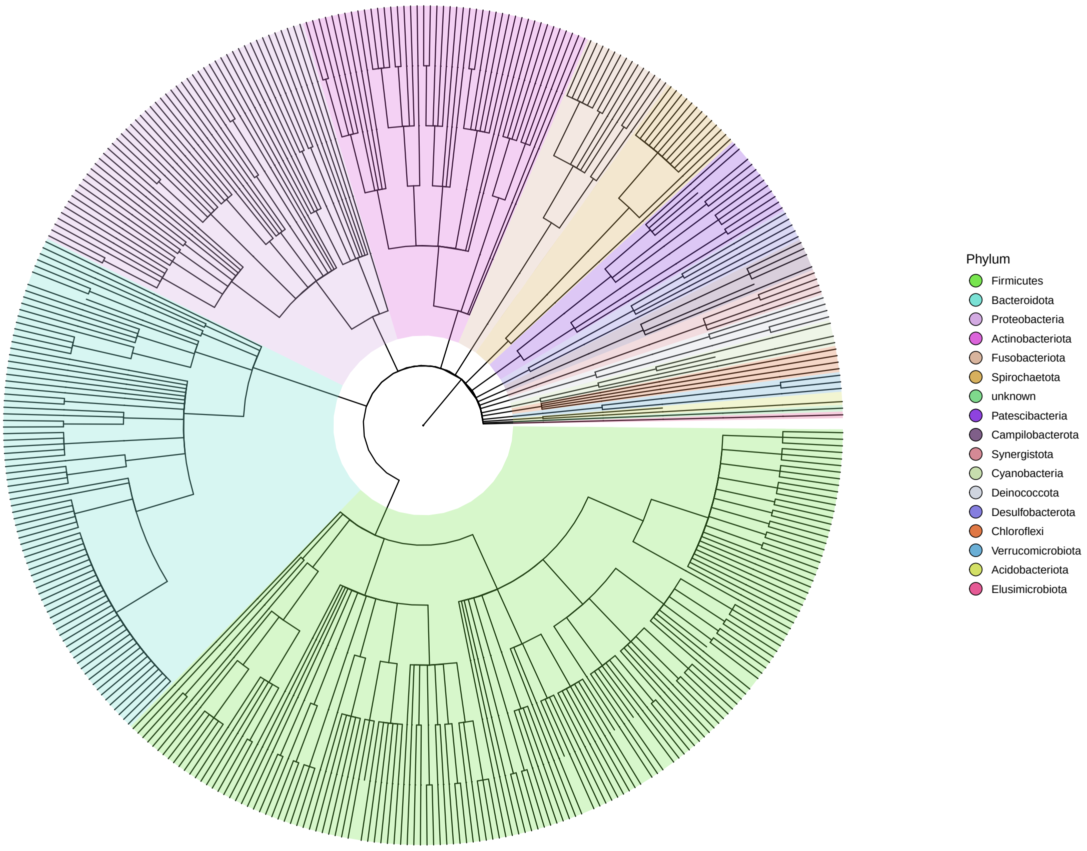

Figure 1: taxononmy structure processed by QIIME 2

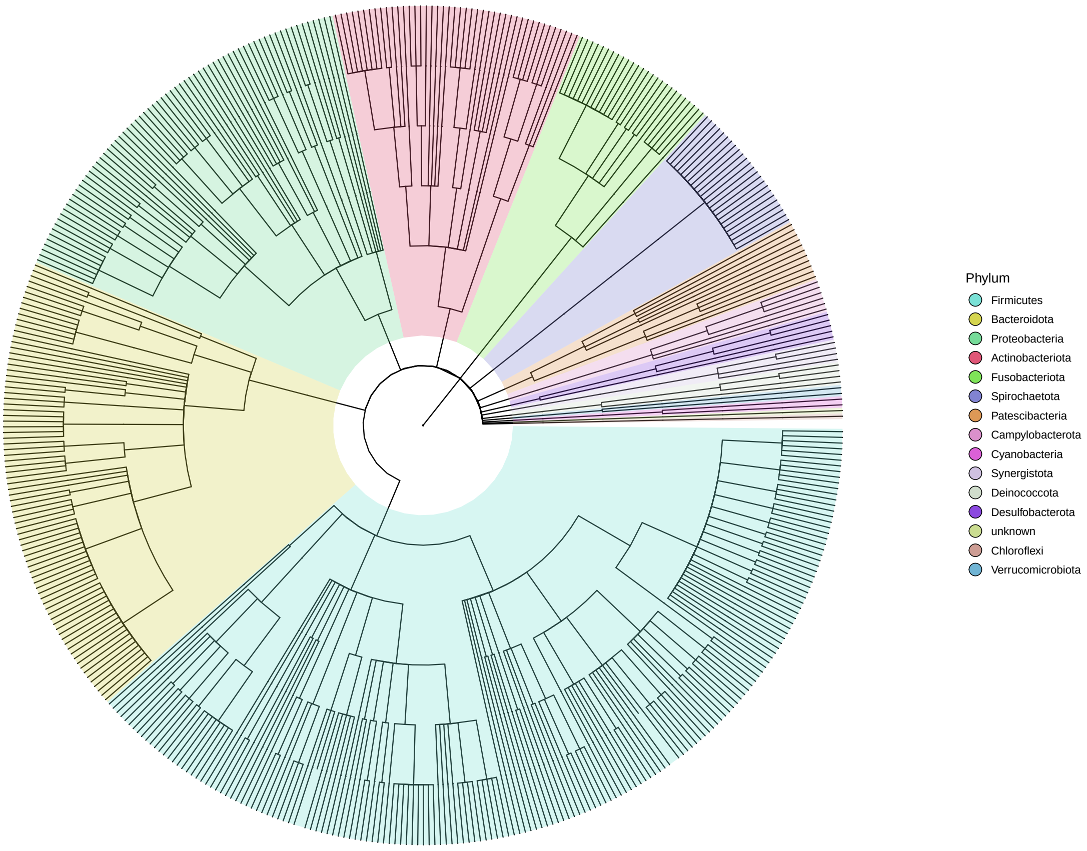

Figure 2: taxononmy structure processed by QIIME 2

Woltka. Both tools were applied to the same whole-genome shotgun (WGS) metagenomic dataset from Qiita [51], specifically study 11808: "Oral Infections, Glucose Intolerance, and Insulin Resistance" [52]. This dataset comprises 181 samples, and our objective was to compare the taxonomic profiling and phylogenetic outputs generated by the two tools.

Since phylogenetic trees are constructed based on the taxa detected by each pipeline, and the relationships among these taxa form the structure of the tree. Therefore, the number of taxa, their distribution across taxonomic ranks, and the overlap between the tools are key factors that shape the overall tree. Table 4 presents the number of taxa detected by MetaPhlAn 4 and Woltka across different taxonomic levels, with the overlap between the two pipelines also listed.

To provide a clearer illustration of the differences in taxonomic resolution, we visualized the taxonomy trees by highlighting the phyla with different colors as illustrate in Figure 3 and Figure 4. The first noticeable difference between MetaPhlAn 4 and Woltka is the number of tree tips, representing the detected taxa. MetaPhlAn 4 identifies fewer features compared to Woltka, resulting in a simpler tree structure. However, both pipelines capture the dominant phyla, such as Firmicutes, Bacteroidetes, and Proteobacteria.

A significant distinction is that Woltka detects a greater number of unclassified phyla, particularly those labeled as "Candidatus," which are often novel or poorly characterized taxa. This reflects Woltka's broader coverage in identifying rare or unclassified lineages, leading to a more complex and detailed phylogenetic tree.

Table 4: Comparison of MetaPhlAn 4 and Woltka across Taxonomic Ranks

	**Phylum**	**Class**	**Order**	**Family**	**Genus**
**MetaPhlAn 4**	11	42	52	65	122
**Woltka**	31	49	117	244	997
**Overlap**	8	15	23	32	47

5 Guide for MetaPhlAn 4

Step 1: Database Installation for MetaPhlAn 4: After installing MetaPhlAn 4, users need to manually install the MetaPhlAn 4 database to ensure proper functionality.

Step 2: Processing Sequencing Files: Use MetaPhlAn 4 to process each data sample individually, ensuring accurate taxonomic profiling.

Step 3: Organizing Results and Mapping to Reference Tree: Since MetaPhlAn 4 outputs results for each sample separately, all results should be merged. Users can then map the SGBs to a reference tree to construct a comprehensive phylogenetic tree.

6 Introduction to Kraken 2

Kraken 2 is developed from Kraken, an ultrafast metagenomic sequence classifier using exact alignments [53]. Kraken 2 addresses the significant resource demands of its predecessor by optimizing the data structure and algorithm. It introduces a probabilistic, compact hash table that maps minimizers to their lowest common ancestors (LCA), significantly reducing memory usage while maintaining high classification accuracy, it also examines k-mers which are substrings of length contained within a biological sequence

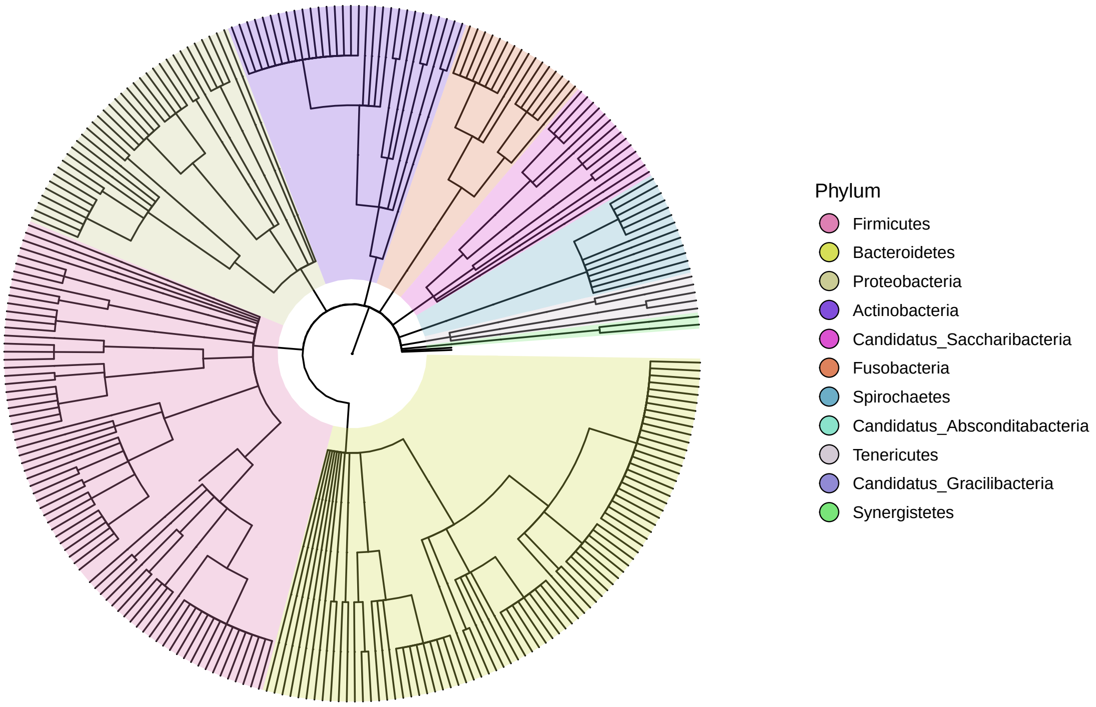

Figure 3: taxonomy structure processed by MetaPhlAn 4

within the query sequence, searches for them in the database, looks for where these are placed within the taxonomy tree inside the database, makes the classification with the most probable position, then maps k-mers to LCA of all genomes known to contain the given k-mer. Kraken 2 accepts a variety of input file formats, including FASTA and FASTQ files, allowing it to handle raw sequence data efficiently. Unlike MetaPhlan 4, which focuses on using unique marker genes for taxonomic profiling, Kraken 2 offers the flexibility to classify sequences using any custom database, including human, bacterial, and other databases. However, it's worth noting that building a phylogenetic tree directly from Kraken 2 results remains an exploratory area, requiring additional steps and tools to integrate classification outputs into phylogenetic analyses.

References

[1] D. E. Wood, J. Lu, B. Langmead, Improved metagenomic analysis with kraken 2, Genome biology 20 (2019) 1–13.

[2] C. Gondro, B. P. Kinghorn, A simple genetic algorithm for multiple sequence alignment, Genetics and Molecular Research 6 (4) (2007) 964–982.

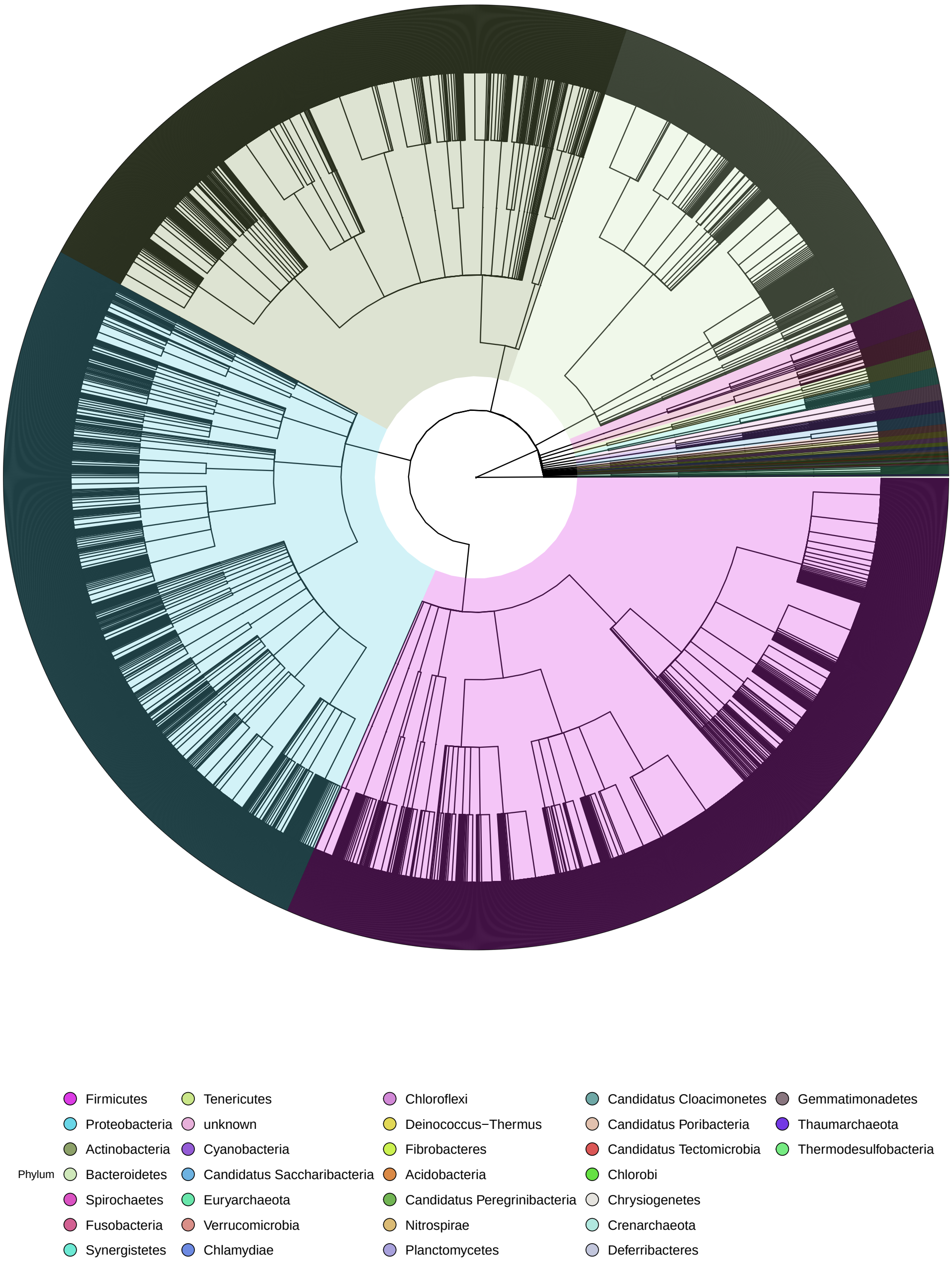

Figure 4: taxononmy structure processed by Woltka

[3] B. Chowdhury, G. Garai, A review on multiple sequence alignment from the perspective of genetic algorithm, Genomics 109 (5-6) (2017) 419–431.

[4] S. B. Needleman, C. D. Wunsch, A general method applicable to the search for similarities in the amino acid sequence of two proteins, Journal of molecular biology

48 (3) (1970) 443–453.

[5] T. F. Smith, M. S. Waterman, et al., Identification of common molecular subsequences, Journal of molecular biology 147 (1) (1981) 195–197.

[6] B. Langmead, S. L. Salzberg, Fast gapped-read alignment with bowtie 2, Nature methods 9 (4) (2012) 357–359.

[7] D. Kim, B. Langmead, S. L. Salzberg, Hisat: a fast spliced aligner with low memory requirements, Nature methods 12 (4) (2015) 357–360.

[8] H. Li, Minimap2: pairwise alignment for nucleotide sequences, Bioinformatics 34 (18) (2018) 3094–3100.

[9] D. A. Morrison, Multiple sequence alignment for phylogenetic purposes, Australian Systematic Botany 19 (6) (2006) 479–539.

[10] J. D. Thompson, B. Linard, O. Lecompte, O. Poch, A comprehensive benchmark study of multiple sequence alignment methods: current challenges and future perspectives, PloS one 6 (3) (2011) e18093.

[11] F. Sievers, A. Wilm, D. Dineen, T. J. Gibson, K. Karplus, W. Li, R. Lopez, H. McWilliam, M. Remmert, J. Söding, et al., Fast, scalable generation of high-quality protein multiple sequence alignments using clustal omega, Molecular systems biology 7 (1) (2011) 539.

[12] K. Katoh, J. Rozewicki, K. D. Yamada, Mafft online service: multiple sequence alignment, interactive sequence choice and visualization, Briefings in bioinformatics 20 (4) (2019) 1160–1166.

[13] S. Henikoff, J. G. Henikoff, Amino acid substitution matrices from protein blocks., Proceedings of the National Academy of Sciences 89 (22) (1992) 10915–10919.

[14] M. Dayhoff, R. Schwartz, B. Orcutt, 22 a model of evolutionary change in proteins, Atlas of protein sequence and structure 5 (1978) 345–352.

[15] S. F. Altschul, A protein alignment scoring system sensitive at all evolutionary distances, Journal of molecular evolution 36 (1993) 290–300.

[16] E. Michu, et al., A short guide to phylogeny reconstruction, Plant Soil and Environment 53 (10) (2007) 442.

[17] R. Godini, H. Fallahi, A brief overview of the concepts, methods and computational tools used in phylogenetic tree construction and gene prediction, Meta Gene 21 (2019) 100586.

[18] P. Sneath, R. Sokal, Numeral taxonomy, WH: Freemam, San Francisco, California (1973).

[19] N. Saitou, M. Nei, The neighbor-joining method: a new method for reconstructing phylogenetic trees., Molecular biology and evolution 4 (4) (1987) 406–425.

[20] T. Bergmann, H. Hadrys, G. Breves, B. Schierwater, Character-based dna barcoding: a superior tool for species classification, Berliner und Münchener Tierärztliche Wochenschrift 122 (11/12) (2009) 446–450.

[21] L. L. Cavalli-Sforza, A. W. Edwards, Phylogenetic analysis. models and estimation procedures, American journal of human genetics 19 (3 Pt 1) (1967) 233.

[22] W. M. Fitch, E. Margoliash, Construction of phylogenetic trees: a method based on mutation distances as estimated from cytochrome c sequences is of general applicability., Science 155 (3760) (1967) 279–284.

[23] R. Davidson, S. Sullivant, Distance-based phylogenetic methods around a polytomy, IEEE/ACM Transactions on Computational Biology and Bioinformatics 11 (2) (2014) 325–335.

[24] Y. Van de Peer, M. Salemi, Phylogenetic inference based on distance methods, The phylogenetic handbook 2 (2009) 142–159.

[25] Y. Van de Peer, M. Salemi, Phylogeny inference based on distance methods, The Phylogenetic Handbook, a Practical Approach to DNA and Protein Phylogeny (2003) 101–136.

[26] T. Stefan Van Dongen, B. Winnepenninckx, Multiple upgma and neighbor-joining trees and the performance of some computer packages, Mol. Biol. Evol 13 (2) (1996) 309–313.

[27] M. K. Kuhner, J. Felsenstein, A simulation comparison of phylogeny algorithms under equal and unequal evolutionary rates., Molecular biology and evolution 11 (3) (1994) 459–468.

[28] A. Rzhetsky, M. Nei, A simple method for estimating and testing minimum-evolution trees, Mol Biol Evol 9 (5) (1992) 945–967.

[29] K. Tamura, G. Stecher, S. Kumar, Mega11: molecular evolutionary genetics analysis version 11, Molecular biology and evolution 38 (7) (2021) 3022–3027.

[30] P. A. Goloboff, J. S. Farris, K. C. Nixon, Tnt, a free program for phylogenetic analysis, Cladistics 24 (5) (2008) 774–786.

[31] S. Mirarab, N. Nguyen, T. Warnow, Sepp: Saté-enabled phylogenetic placement, in: Biocomputing 2012, World Scientific, 2012, pp. 247–258.

[32] J. Felsenstein, Evolutionary trees from dna sequences: a maximum likelihood approach, Journal of molecular evolution 17 (1981) 368–376.

[33] T. H. Jukes, C. R. Cantor, et al., Evolution of protein molecules, Mammalian protein metabolism 3 (24) (1969) 21–132.

[34] M. Kimura, A simple method for estimating evolutionary rates of base substitutions through comparative studies of nucleotide sequences, Journal of molecular evolution 16 (1980) 111–120.

[35] K. Tamura, M. Nei, Estimation of the number of nucleotide substitutions in the control region of mitochondrial dna in humans and chimpanzees., Molecular biology and evolution 10 (3) (1993) 512–526.

[36] M. Hasegawa, H. Kishino, T.-a. Yano, Dating of the human-ape splitting by a molecular clock of mitochondrial dna, Journal of molecular evolution 22 (1985) 160–174.

[37] S. Tavaré, Some probabilistic and statistical problems on the analysis of dna sequence., Lecture of Mathematics for Life Science 17 (1986) 57.

[38] M. N. Price, P. S. Dehal, A. P. Arkin, Fasttree 2–approximately maximum-likelihood trees for large alignments, PloS one 5 (3) (2010) e9490.

[39] A. Stamatakis, Raxml-vi-hpc: maximum likelihood-based phylogenetic analyses with thousands of taxa and mixed models, Bioinformatics 22 (21) (2006) 2688–2690.

[40] L.-T. Nguyen, H. A. Schmidt, A. Von Haeseler, B. Q. Minh, Iq-tree: a fast and effective stochastic algorithm for estimating maximum-likelihood phylogenies, Molecular biology and evolution 32 (1) (2015) 268–274.

[41] S. Guindon, O. Gascuel, A simple, fast, and accurate algorithm to estimate large phylogenies by maximum likelihood, Systematic biology 52 (5) (2003) 696–704.

[42] J. Felsenstein, Phylogenies from molecular sequences: inference and reliability, Annual review of genetics 22 (1) (1988) 521–565.

[43] Z. Yang, Computational molecular evolution, OUP Oxford, 2006.

[44] B. Rannala, Z. Yang, Probability distribution of molecular evolutionary trees: a new method of phylogenetic inference, Journal of molecular evolution 43 (1996) 304–311.

[45] Z. Yang, B. Rannala, Bayesian phylogenetic inference using dna sequences: a markov chain monte carlo method., Molecular biology and evolution 14 (7) (1997) 717–724.

[46] Z. Yang, B. Rannala, Molecular phylogenetics: principles and practice, Nature reviews genetics 13 (5) (2012) 303–314.

[47] B. Larget, D. L. Simon, Markov chain monte carlo algorithms for the bayesian analysis of phylogenetic trees, Molecular biology and evolution 16 (6) (1999) 750–759.

[48] A. J. Drummond, A. Rambaut, Beast: Bayesian evolutionary analysis by sampling trees, BMC evolutionary biology 7 (2007) 1–8.

[49] N. Lartillot, T. Lepage, S. Blanquart, Phylobayes 3: a bayesian software package for phylogenetic reconstruction and molecular dating, Bioinformatics 25 (17) (2009) 2286–2288.

[50] F. Ronquist, J. P. Huelsenbeck, Mrbayes 3: Bayesian phylogenetic inference under mixed models, Bioinformatics 19 (12) (2003) 1572–1574.

[51] A. Gonzalez, J. A. Navas-Molina, T. Kosciolek, D. McDonald, Y. Vázquez-Baeza, G. Ackermann, J. DeReus, S. Janssen, A. D. Swafford, S. B. Orchanian, et al., Qiita: rapid, web-enabled microbiome meta-analysis, Nature methods 15 (10) (2018) 796–798.

[52] R. Demmer, D. Jacobs Jr, R. Singh, A. Zuk, M. Rosenbaum, P. Papapanou, M. Desvarieux, Periodontal bacteria and prediabetes prevalence in origins: the oral infections, glucose intolerance, and insulin resistance study, Journal of dental research 94 (9_suppl) (2015) 201S–211S.

[53] D. E. Wood, S. L. Salzberg, Kraken: ultrafast metagenomic sequence classification using exact alignments, Genome biology 15 (2014) 1–12.
